# Neither caloric nor protein restriction increases the male lifespan of outbred short-lived fish

**DOI:** 10.1093/gerona/glaf278

**Published:** 2025-12-13

**Authors:** Jakub Žák, Martin Reichard

**Affiliations:** Department of Botany and Zoology, Faculty of Science, Masaryk University, Brno, Czech Republic; Department of Botany and Zoology, Faculty of Science, Masaryk University, Brno, Czech Republic; Institute of Vertebrate Biology, Czech Academy of Sciences, Brno, Czech Republic; (Biological Sciences Section)

**Keywords:** Animal model, Biology of aging, Nutrition, Turquoise killifish

## Abstract

Laboratory inbred strains respond strongly to dietary restriction (DR), whereas genetically diverse populations may not experience comparable benefits. The exceptionally short-lived fish, *Nothobranchius furzeri*, offers several genetically distinct captive populations; however, only the severely inbred GRZ strain has been tested for DR effects. Here, individually kept males (*N* = 111) of the outbred MZCS 222 strain of *N. furzeri* were assigned to 1/high-protein diet and two DR forms: 2/low-protein (isocaloric to high-protein but half the protein concentration), or 3/caloric-restriction (half the dose of HP at the feeding time). Neither form of DR significantly extended the lifespan of *N. furzeri* males. The limited efficacy of protein and caloric restriction in outbred *N. furzeri* may stem from insensitivity to these forms of DR or from the genetic diversity of the strain in comparison to earlier reported life extension through intermittent fasting in the inbred GRZ strain.

## Introduction

Dietary restriction has a life-extending effect on diverse taxa.^1^ It supposedly acts through increased somatic maintenance and temporal postponement of growth, which, in accordance with life history trade-off, extends lifespan.^2^^,^^3^ Although life extension is intensified by caloric restriction in rats (*Rattus norvegicus*), the lifespan of threespine stickleback fish (*Gasterosteus aculeatus*) responds to protein but not caloric restriction.^4^^,^^5^ Therefore, recent attention has shifted from caloric restriction to restricting specific nutrients, emphasizing a comparative approach among various dietary restriction forms to disentangle their true impact.

The effects of dietary restriction are not always positive.^6^^,^^7^ In traditional aging models, population-specific responses to dietary restriction are common, with some populations even exhibiting shorter lifespans.^6^^,^^7^ This variability was attributed to genetic predisposition and to the level of adaptation to captivity, as captive-adapted strains were more sensitive to DR.^1^^,^^8^^,^^9^ Given the inconsistent effects of dietary restriction, the use of less-studied species is welcome, especially when populations with different genetic backgrounds and breeding histories are available.^10^

Turquoise killifish *Nothobranchius furzeri* is an extremely short-lived fish with a maximum lifespan of several months.^11^ Its rapid aging, easy captive breeding, and various populations with different natural lifespans made it a promising research organism in biogerontology.^12^ Intermittent fasting, where caloric intake is deprived by less frequent feeding and consequent prolonged fasting periods, was reported to extend the lifespan of the inbred laboratory GRZ strain,^13-15^ but finer manipulation of its diet composition has not been possible due to the lack of appropriate synthetic feed. The inbred GRZ strain has been kept in captivity for over 50 years, and many of its life history traits differ from outbred strains.^14^^,^^16^ The limited data from a single strain hinders our ability to draw firm conclusions about the impact of dietary restrictions on this model species.

Here, we subjected individually kept males of the outbred *N. furzeri* MZCS 222 strain to caloric and protein restriction. In contrast to previous *N. furzeri* DR studies,^13-15^ the caloric restriction was induced by halving doses at feeding time and not by intermittent fasting. Protein restriction, never studied in *N. furzeri*, was induced by feeding with a controlled dose of a synthetic protein-deprived isocaloric diet. We hypothesized that dietary restriction would decrease the growth of the fish and significantly extend lifespan, as seen in the GRZ strain. Covering the two unstudied forms of dietary restriction in *N. furzeri* would provide fundamental insights into mechanisms responsible for aging interventions using this model species.

## Methods

### Housing

*Nothobranchius furzeri* males (*N* = 111) of the outbred MZCS 222 strain^11^ were, from the age of 8 weeks, housed individually within one recirculating system with 2 L plastic compartments at the Institute of Vertebrate Biology, CAS. The system water was maintained at a conductivity of 1.5 mS × cm^−2^, temperature at 26.5 °C (range 25-28 °C), and the light regime was 14 L:10D. The recirculating system contained five sentinel fish, which were histologically examined at 10, 13, 24, 47, and 53 weeks of age; all were pathogen-free, thereby excluding the effect of background infection on fish mortality.

### Experimental diets

Adults from the age of 9 weeks fully accepted synthetic diet and were split into three dietary groups (37 males each); 1/High Protein group (HP: 67% proteins-P, 8% lipids-L, Table 1), 2/Low Protein group (LP: 33% P, 22% L, Table 1), and 3/Caloric Restriction group (half the dose of the HP diet at each feeding). High-protein and low-protein diets were isocaloric by increasing the fish oil (lipid) ratio in the LP diet (Table 1). Full details regarding the formula are at Žák and Vrtílek.^17^ To have full control over caloric and nutrient intake, fish were fed a fixed dose (dispensed by Lee Perfect Powder Measure, Hartford, Wisconsin) twice daily, at least 6 h apart. Ad libitum feeding could lead to undesirable compensatory feeding of fish fed by less nutritionally dense feed.^18^ The optimal feed dose was reassessed every two weeks and increased when less than half of the HP group had some uneaten feed 5 min after afternoon feeding. The daily dose of feed in HP and LP groups ranged from 20 to 42 mg, depending on the fish age. Body mass at the start of the experiment did not differ among groups (ANOVA, *F*_2,80_ = 0.65, *p* = .524).

**Table 1.** The formula of purified diets used for the dietary restriction experiment using *Nothobranchius furzeri* MZCS 222.

	High protein	Low protein
Ingredient	Per 100 g	Per 100 g
**Casein**	58.05	30.40
**Albumin**	11.45	6.00
**Lecithin**	3.00	3.00
**Menhaden fish oil**	8.00	22.30
**Dextrin**	8.00	8.00
**Alginate**	2.00	2.00
**Ascorbic palmitate**	0.70	0.70
**Betaine**	0.20	0.20
**Mineral mix^a^**	5.00	5.00
**Vitamin mix** ^b^	1.60	1.60
**Microcrystalline cellulose**	2.00	20.80
**Expected protein and lipid gross energy^c^ (kcal × 100 g^−1^)**	327	334

Macronutrient	High protein	Low protein
**Crude protein (%)**	66.68	33.94
**Crude fat (%)**	8.23	21.65
**Protein:Lipid ratio**	8.1:1.0	1.6:1:0
**Analytic gross protein and lipid energy^c^ (kcal × 100 g^−1^)**	340	331

Macronutrients are expressed as percentages of the dry matter. Carbohydrates were not analyzed as they have low importance in the energy intake of mesopredators, such as *N. furzeri*. See (17) for more details about diets.

a See Žák and Vrtílek.^17^

b See Žák and Vrtílek.^17^

c Only protein and lipid energy are taken into consideration, as these are the only nutrients that vary between purified diets. Others were constant. In addition, these are the most metabolically important macronutrients in teleost fish.

### Sampling

Fish survival was recorded daily. Body mass (the wet mass of live fish, with a precision of 0.001 g by Kern PCB 350-3) and body size (total length, TL 1 mm precision) were recorded once per month. Body condition was computed as residuals of log(body mass) – log(TL) regression, with higher values denoting greater body condition.

### Ethics

All procedures followed institutional (Nr. 62116/2017-MZE-17214), national (CZ laws 246/1992 and 419/2012), and international (EU Directive 2010/63/EU) legislation.

### Statistical analysis

Diet-dependent survival (*N* = 111) was compared using the Cox Proportional Hazard (CoxPH) model from the *survival* package v 3.7.0. The post-hoc power test and expected sample size to find a significant difference were assessed by powerCT and sampleCT commands from the package powerSurvEpi v.0.1.3 for each dietary (pairwise) contrast separately. Parametric instantaneous mortality hazard ratio was assessed by survival regression with Weibull distribution, and mortality hazards were predicted using *eha* package. Terzibasi et al.^14^ reported that the dietary restriction effect was intensified in long-lived adults; therefore, the CoxPH with individuals surviving longer than the median lifespan (42 weeks, *N* = 63) was repeated. Terminal 10% survivorship was tested by a pairwise comparison with the HP diet using a *z*-test computed from diet-specific estimates produced by *survfit* at the age of 427 days, when 10% of survivors remained in the HP group.

Diet-specific change in body condition with age (*N* = 530) was analyzed by Gaussian generalized additive models (GAMs) and four knots (*k* = 4), using mgcv v 1.9.-1 package. The growth in body mass trajectory was fitted using GAMs with *k* = 5. Diet-specific curves were fitted. Only data (*N* = 542), until at least three individuals per diet were available (age of 472 days), were used. In all GAMs, individual fish ID was treated as a random effect.

Decline in body mass at advanced age (from the age of 48 weeks, *N* = 78) was analyzed by linear mixed effect model (LME) with fish ID as a random factor using *lme4* package v1.1-35.5. The full model included age–diet interaction, which was removed as it was not significant (*p* > .05). All statistical analyses were performed in R version 4.4.2. Data supporting the study findings are at FigShare repository (doi: 10.6084/m9.figshare.29896151).

## Results

**Figure 1.**
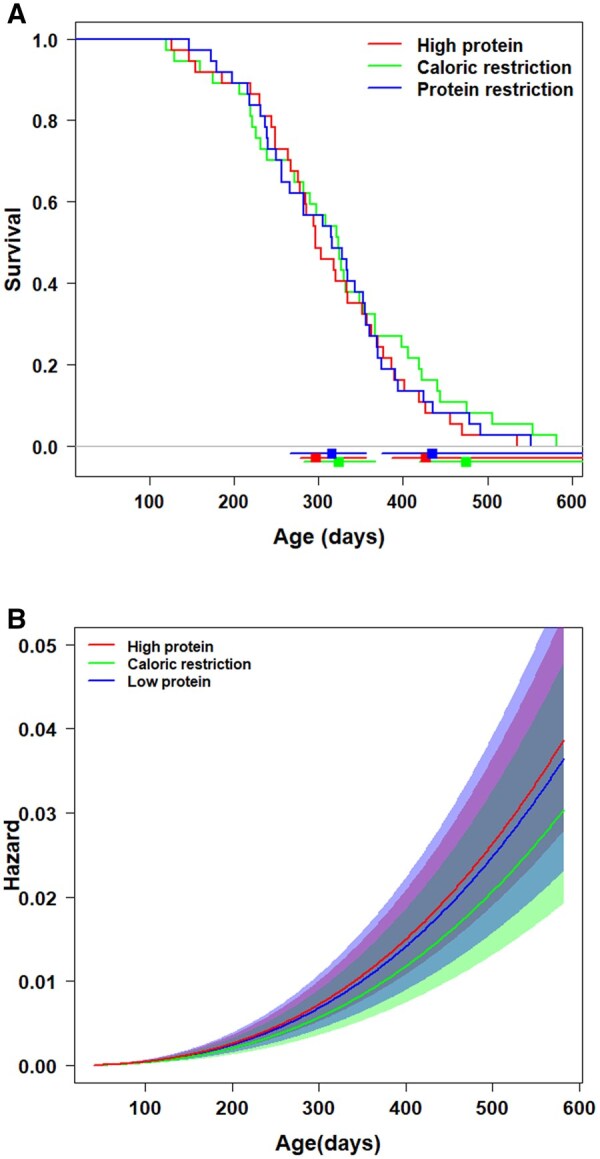
The effect of protein and caloric restriction on survival and mortality rate of *N. furzeri* males from an outbred MZCS 222 strain. (A) Survival is unaffected by the protein (blue) and caloric (green) restriction. Kaplan-Meier plot (*N* = 111). Square points with error bars at the bottom of the plot are medians and 10% survival with 95% CI. CIs are estimated from the Kaplan-Meier plot. (B) Age-dependent instantaneous mortality hazard from the parametric Weibull model demonstrates a monotonic increase irrespective of dietary treatment. The shaded areas around curves in (B) are a 95% CI.

Neither caloric (CoxPH, hazard ratio (HR) = 0.80, SE = 0.24, *z* = -0.92, *p* = .357) nor protein restriction (HR = 0.95, SE = 0.23, *z* = -0.23, *p* = .819) affected survival when compared to the high protein diet (Figure 1A). The parametric Weibull model confirmed strong overlap among trajectories of dietary-specific instantaneous mortality hazards (Figure 1B). Lifespan was comparable even for long-lived individuals (χ^2^_2_ = 1.13, *p* = .569). Similarly, 10% survival was not diet dependent (HP vs LP, *z* = -0.40, *p* = .691, HP vs CR, *z* = -1.07, *p* = .282). The sample size required to observe a significant difference between HP and CR (observed power 0.507) would be 370 individuals, and between HP and LP (observed power 0.501), 3544 individuals.

**Figure 2.**
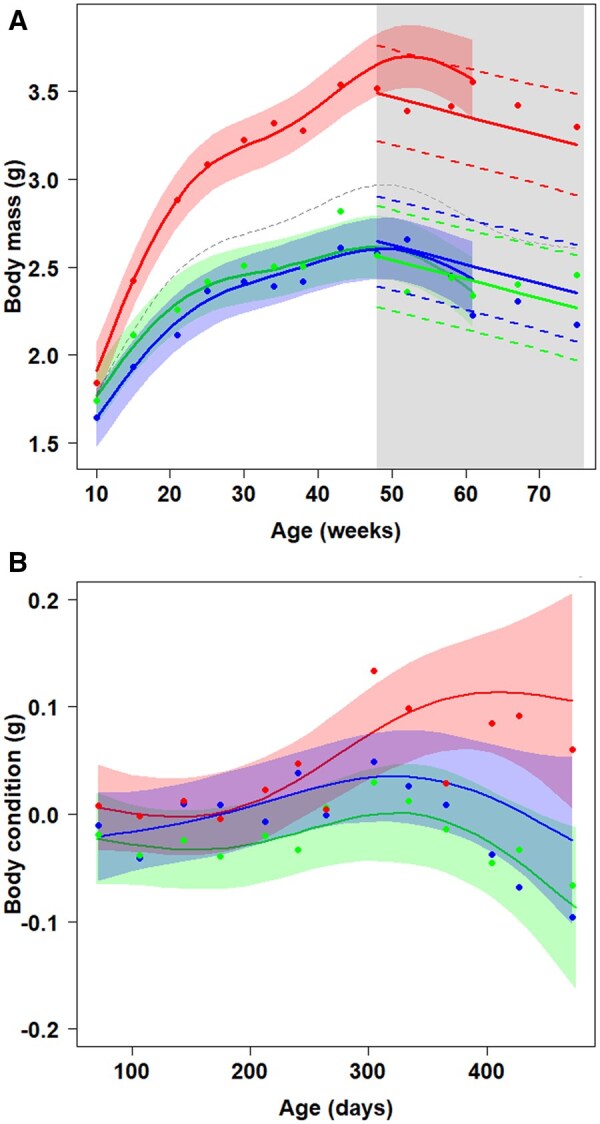
The effect of protein and caloric restriction on growth in body mass, terminal decline in body mass, and trajectory of body condition throughout the lifespan of *N. furzeri* males from an outbred MZCS 222 strain. (A) Age-dependent body mass trajectory showing a decline in body mass at advanced age (from 48 weeks on). The gray area represents the age when decline is observed. The dashed black curve represents the average trend, regardless of the dietary treatment used, to determine the age at which body mass began to decline. Shaded confidence intervals around the growth curves are only for data where at least three males per diet were still alive. Straight lines are trends of terminal decline in the body mass estimated by a linear mixed-effect model. Dashed lines for these trends delimit 95% CI. (B) Age-dependent body condition in relation to diet. High-protein-fed males (red) reached an asymptote, whereas protein-restricted males (blue) exhibited a significant decline in body condition at advanced age. There was no significant change in body condition throughout the lifespan of the caloric-restricted males (green). Points in (A) and (B) are raw means acquired from time intervals of the sampling, and their position may slightly differ from curvature as they are not corrected for the random effect of the repeated measures of the same individual.

Both dietary restrictions decreased fish growth. Body mass of high-protein males was consistently higher (GAM, HP vs CR estimate = 0.64, SE = 0.11, *t* = 5.8, *p* < .001; HP vs LP, estimate = 0.71, SE = 0.11, *t* = 6.36, *p* < .001) but there was no difference between LP and CR males (Figure 2A). Since the age of 48 weeks, body mass declined at an estimated rate of 10 mg per week (SE = 2 mg; LME, χ^2^_1_ = 21.91, *p* < .001), irrespective of the dietary treatment (diet: age interaction removed, χ^2^_2_ = 5.93, *p* = .055, Figure 2A).

Age-dependent dynamics in body condition was also affected by the diet. High protein-fed fish condition increased until reaching an asymptote (GAM, edf = 2.67, ref.df = 2.91, *F* = 8.79, *p* < .001). Caloric-restricted fish had a stable body condition throughout their lifespans, with a non-significant decrease at senescent age (edf = 2.88, ref.df = 2.97, *F* = 1.75, *p* = .137). Protein-restricted males experienced a significant decline in body condition at advanced age (edf = 2.50, ref.df = 2.81, *F* = 2.77, *p* = .028, Figure 2B).

## Discussion

Using a purified diet, we found that neither caloric nor protein restriction affected the lifespan of an outbred *N. furzeri* strain. Still, both dietary restrictions slowed fish growth, which is generally assumed to lead to lifespan extension.^3^ Irrespective of the diet, fish exhibited senescence in terms of terminal decline in body mass, a common vertebrate aging phenotype. Our study highlights low susceptibility to lifespan extension of males of the outbred *N. furzeri* MZCS 222 strain by protein and caloric restriction, despite previous reports on lifespan extension by intermittent fasting in the inbred *N. furzeri* GRZ strain.^13-15^

Clear differences in body mass and body condition among the treatments were not associated with lifespan, despite the commonly observed association in other species.^3^ Previously, growth retardation by temperature was significantly associated with longer lifespan in various strains of *N. furzeri*^19^^,^^20^ but no relationship between growth and lifespan was found elsewhere.^16^ This inconsistency suggests a possibility of a context-specific lifespan-body size relationship and requires further investigation.

It is possible that *N. furzeri* is one of the few less-sensitive species to protein restriction, such as rats.^4^ Also, fasting, rather than caloric restriction *per se*, seems to be the crucial component for life extension.^21^ Here, caloric restriction was induced by halving feed portions, reducing the fasting period compared to previous *N. furzeri* intermittent fasting studies, where longer fasting (but similar calorie reduction, ie, by 50%^14^ and 57%^13^) enhanced longevity.^13^^,^^14^ Although inconclusive, this suggests *N. furzeri* may respond to intermittent fasting rather than to caloric or protein restriction.

Choosing fats over carbohydrates to have isocaloric HP and LP diets may have impacted the absence of protein restriction lifespan benefits, as demonstrated in laboratory mice, where low-protein–high-carbohydrate diets extended lifespan more than low-protein–high-fat diets.^22^ Nonetheless, fats are more metabolically critical than carbohydrates for fish, which contrasts with mice.^23^ Thus, increased carbohydrates may not contribute metabolically to fish caloric intake, potentially acting as an undigestible diluent. Despite the solid feeding design, the *N. furzeri* did not experience lifespan extension when dietary restricted.

Synthetic diets are sometimes criticized for shortening the lifespans of test subjects.^8^ Here, the median lifespan of 10 months and a maximum lifespan above 18 months is in the upper range of lifespan records for this species and strain. This demonstrates that our study fish did not suffer from malnutrition and successfully adopted a synthetic diet. We do not think that the long lifespan observed in all our dietary treatments was caused by unintentionally inducing DR-like conditions in all treatments (including HP), as a similar long lifespan of this strain has been reported in earlier studies with ad libitum-fed fish.^19^^,^^24^

The strict use of males in our study may be a contributing factor to the absence of the lifespan-extending effect of dietary interventions. Females generally respond strongly to dietary restriction due to their higher lifespan-reproduction trade-off.^1^ However, an earlier study showed that inbred *N. furzeri* males are more affected by DR than females.^13^ Therefore, the use of males has its own validity in this research organism, while we acknowledge that it prevents generalization to females.

Low power of the study design could also result in the lack of detection of the lifespan-extending effect. The study of intermittent fasting of the GRZ strain had a limit of 12 individuals for the survival difference detection (recalculated from Terzibasi et al.^14^). In the same study, 90 individuals of outbred MZM 0410 were insufficient for detecting lifespan differences among dietary regimes, and a sample size of 428 individuals would be required to demonstrate differences between the treatments.^14^ A comparable sample size would be required in our study to detect a difference between a high-protein diet and a caloric-restricted diet. An order of magnitude higher sample size would be required to detect the lifespan extension of the low-protein diet. The differences between control and treatments in our study were too low to suggest any biologically relevant contrast, and we believe that our study does not fail to provide a positive effect due to low statistical power.

The insensitivity to protein and caloric restriction is not likely the consequence of a lack of adaptation to laboratory conditions (as reported by Lee et al.^8^ and Harper et al.^9^) because our study strain has been kept in captivity for more than a decade,^11^ long enough to be adapted. Instead, the genetic diversity or background of the strain may play a role.^7^^,^^25^ The fact that lifespan extensions in *N. furzeri* by intermittent fasting are reported only for the inbred laboratory strain^13-15^ supports the major role of genetic architecture.

## Conclusion

The first successful use of the synthetic diet for *N. furzeri* allowed us to manipulate diet composition and test the effect of caloric and protein restrictions on lifespan. We found that either restriction had no effect on the lifespan of the outbred MZCS 222 strain. This reaffirms that the relationship between dietary intervention and lifespan is not straightforward, and results observed in one strain may not apply to others. The genetic background and diversity of the outbred strain could have contributed to the lack of response to dietary restrictions.^25^ Also, intermittent fasting may simply be a more efficient aging intervention in the *N. furzeri*, especially when an inbred strain is used.

## Data Availability

Data supporting the study findings can be found at FigShare repository 10.6084/m9.figshare.29896151.
